# Evaluation of lipoprotein-associated Phospholipase A2 in Healthy Chinese Han Adult Serum

**DOI:** 10.1186/1476-511X-13-6

**Published:** 2014-01-07

**Authors:** Li-Min Feng, Guo-Fang Feng, Yu Chen

**Affiliations:** 1 Department of Laboratory Medicine, The First Affiliated Hospital, College of Medicine, Zhejiang University, Hangzhou 310003, P.R. China; 2 Department of Reproductive Endocrinology, Women’s Hospital, School of Medicine, Zhejiang University, Hangzhou 310006, P.R. China

**Keywords:** Lipoprotein-associated phospholipase A2 (Lp-PLA2), Chinese Han adult, Reference interval

## Abstract

**Objective:**

The aim of this study is to establish lipoprotein-associated phospholipase A2 (Lp-PLA2) reference intervals (RIs) in healthy Chinese Han adults as a clinical diagnostic indicator according to the Clinical and Laboratory Standards Institute (CLSI) C28-A3 guidelines.

**Design and methods:**

Lp-PLA2 levels in 763 healthy Chinese Han subjects (392 males and 371 females) were determined by colorimetric analysis and the central 95th percentile RIs were determined using non-parametric statistical methods. The correlations between serum Lp-PLA2 and blood markers were analyzed by Spearman correlation analyses.

**Results:**

The Lp-PLA2 levels showed a Gaussian distribution with a statistically significant difference between females and males (t = 4.866, P < 0.001). The RIs of serum Lp-PLA2 were 194–640 U/L (18–49 years) and 208–698 U/L (50–88 years) for females, and 230–728 U/L for males. There was a positive correlation between Lp-PLA2 levels and age, Body Mass Index (BMI), as well as with levels of alanine aminotransferase (ALT), gamma-glutamyltransferase (GGT), total bilirubin (TBIL), triglyceride (TG), total cholesterol (Tch), low density lipoprotein cholesterol (LDL-c), apolipoprotein B (apoB), glucose (Glu), high sensitivity C reactive protein (Hs-CRP), white blood cell (WBC), hemoglobin (HGB) and red blood cell (RBC) (P < 0.05). A negative correlation was found with high density lipoprotein cholesterol (HDL-c) and Apolipoprotein AI (apoAI), and no correlation was found with platelet (Plt) levels.

**Conclusion:**

Our results establish the RIs of serum Lp-PLA2 in healthy Chinese Han adults and demonstrate correlations between serum Lp-PLA2 and age, BMI, ALT, GGT, TBIL, TG, Tch, HDL-c, LDL-c, apoAI, apoB, Glu, Hs-CRP, WBC, RBC, and HGB levels.

## Introduction

Lipoprotein-associated phospholipase A2 (Lp-PLA2), also known as plasma platelet-activating factor acetylhydrolase (PAF-AH), is a Ca^2+^-independent enzyme and a member of the phospholipase A2 superfamily [1]. Lp-PLA2 hydrolyzes platelet-activating factor (PAF) and oxidizes phospholipids with a modified short fatty acyl chain esterified at the Sn-2 position [2]. Many studies have indicated that Lp-PLA2 is an independent predictor for cardiovascular disease (CAD), with elevated Lp-PLA2 activity associated with an increased risk for CAD [3-5]. Additionally, Lp-PLA2 levels have been implicated in atherosclerotic plaque formation [6], inflammatory bowel disease [7], acute respiratory distress syndrome [8] and severe anaphylaxis [9].

Brilakis *et al*. [10] revealed that Lp-PLA2 activity and mass are independently influenced by race and gender. The largest ethnic group both in China and the world is the Chinese Han, and therefore related studies investigating the activity distribution of Lp-PLA2 in this population will be of great significance and pave the way for clinical diagnosis and study. Reference intervals of Lp-PLA2 levels are an important parameter for the clinical evaluation of patient health. To the best of our knowledge, there have been no relevant published studies defining Lp-PLA2 reference intervals (RIs) in the Han population of China. This study establishes the RIs according to the recommendations of the CLSI C28-A3 document and evaluates the correlations between serum Lp-PLA2 and blood markers in healthy Han adults [11].

## Materials and methods

### Study population

In this study, 763 adults of Han ethnicity from Zhejiang province in Eastern China who were attending their annual health examination in the healthcare center of the First Affiliated Hospital of Zhejiang University were recruited as subjects. The health status of these participants was determined by a self-report questionnaire. Of the 763 subjects, 392 were males between the ages of 18–87 years (mean: 57.4 years) and 371 were females between 18–88 years (mean: 53.0 years). All participants met the following requirements: no history of cardiovascular disease or diabetes mellitus, not taking lipid-lowering drugs, not taking corticosteroids, triglyceride level below 1.70 mmol/L, total cholesterol level below 5.70 mmol/L, low density lipoprotein cholesterol (LDL-c) level below 3.61 mmol/L, high density lipoprotein cholesterol (HDL-c) above 0.91 mmol/L, fasting blood glucose level below 6.16 mmol/L and normal liver and kidney function. The standards above, excluding populations manifesting abnormal blood lipids that are an important risk factor for atherosclerotic cardiovascular disease, are outlined in the ‘Guidelines on Prevention and Treatment of Blood Lipid Abnormality’ established by the Special Group on Prevention and Treatment of Blood Lipid Abnormality affiliated with the Chinese Journal of Cardiology [12]. This study was approved by the Ethics Committee of the First Affiliated Hospital, College of Medicine, Zhejiang University, and written informed consent was obtained from each individual.

### Sample collection

Venous blood was collected from subjects in a state of quiet and fasting. Serum samples were obtained by centrifugation within 2 hours of initial collection, and stored at - 80°C for processing. Routine analyses of blood samples were completed on the same day.

### Lp-PLA2 assay

Serum samples were tested using a Hitachi 7600 automatic biochemical analyzer (Hitachi Ltd., Tokyo, Japan) equipped with an Azwell Auto PAF-AH kit (Azwell Inc., Osaka, Japan). According to colorimetry methods previously reported by Kosaka *et al.*[13], the activity of Lp-PLA2 was detected based on the principle that it catalytically hydrolyzes the Sn-2 position of the substrate 1-myristoyl-2-(4-nitrophenylsuccinyl) phosphatidylcholine and produces 4-nitrophenyl succinate which is immediately degraded to 4-nitrophenol in an aqueous solution, which can be detected at an absorbance reading of 405 nm.

### Biochemical and blood cell count assays

Alanine aminotransferase (ALT), gamma-glutamyltransferase (GGT), total bilirubin (TBIL), triglyceride (TG), total cholesterol (Tch), high density lipoprotein cholesterol (HDL-c), low density lipoprotein cholesterol (LDL-c), apolipoprotein AI (ApoAI), apolipoprotein B (apoB), glucose (Glu), and high sensitivity C reactive protein (Hs-CRP) levels were measured with a Hitachi 7600 automatic biochemical analyzer using Roche reagents (Roche Diagnostics, Mannheim, Germany) for ALT, GGT, TBIL, TG, Tch and Glu, and using SSUF reagents (SSUF, Shanghai, China) for Hs-CRP, LDL-c, HDL-c, apoAI and apoB. White blood cell (WBC), red blood cell (RBC), and platelet (Plt) levels were measured using the XE-2100 automated hematology analyzer (Sysmex, Kobe, Japan) with Sysmex reagents (Sysmex, Kobe, Japan).

### Statistical analysis

All the experimental data was statistically analyzed using SPSS 13.0 software. The reference intervals acquired are expressed as medians and percentiles. Statistical methods recommended by the CLSI C28-A3 document were used to define the RIs [11]. The recommended nonparametric central 95th percentile intervals were used to define the RIs for their simplicity and reliability [11]. One-sample Kolmogorov-Smirnov tests were used for assessing normality of Lp-PLA2 level distributions and one-way analyses of variance (ANOVA) were used for comparisons across different groups. Outliers in the selected data were detected by means of the Dixon outlier range statistic [11,14]. For comparison between gender groups, Student’s *t*-tests were used, and Spearman correlation analyses were applied for correlations between serum Lp-PLA2 and other blood markers in the whole study population. As an index to estimate the extent of obesity, the body-mass index (BMI) was calculated by body weight (kg) divided by the square of the height (m^2^). For all analyses, a P value below 0.05 indicated statistical significance.

## Results

### Assessment of distribution normality

**Figure 1:**
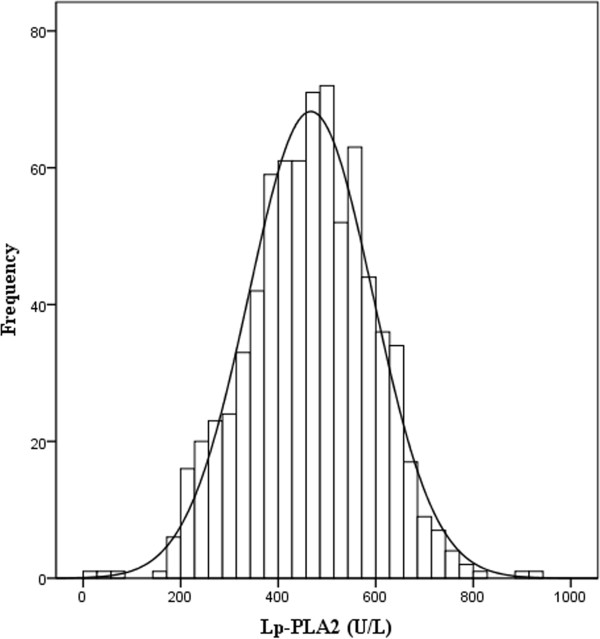
**Gaussian distribution of Lp-PLA2 results.** A one-sample Kolmogorov–Smirnov test of normality indicates a Gaussian distribution.

Table 1 showed the main characteristics of all 763 healthy Chinese Han participants. The distribution of serum Lp-PLA2 levels in all 763 subjects was shown in Figure 1, and the frequency profile illustrated a Gaussian distribution (Z = 0.485, P = 0.972). When a separate statistical analysis was performed by gender, a Gaussian distribution for Lp-PLA2 was also manifested for each gender (male, Z = 1.197, P = 0.114; female, Z = 0.721, P = 0.675).

**Table 1:** Main characteristics of 763 healthy Chinese Han participants

**Variable**	
Age (years)	55.3 ± 15.1
Female/male	371/392
BMI (kg/m^2^)	22.6 ± 3.0
ALT (U/L)	18.6 ± 8.7
GGT (U/L)	23.2 ± 11.0
TBIL (μmol/L)	13.2 ± 4.9
TG (U/L)	1.07 ± 0.41
TCh (U/L)	4.21 ± 0.64
HDL-c (mmol/L)	1.31 ± 0.32
LDL-c (mmol/L)	2.44 ± 0.52
ApoAI (mmol/L)	1.19 ± 0.28
ApoB (mmol/L)	0.72 ± 0.16
fasting glucose (mmol/L)	4.79 ± 0.55
WBC (×10^9^/L)	5.84 ± 1.47
HGB (g/L)	136.6 ± 23.1
RBC (×10^12^/L)	4.42 ± 0.76
Plt (×10^9^/L)	202 ± 59
Hs-CRP (mg/L)	1.7 ± 3.9
Lp-PLA2 activity (U/L)	467 ± 127

*Abbreviations*: *ALT* alanine aminotransferase, *ApoAI* apolipoprotein AI, *ApoB* apolipoprotein B, *BMI* body mass index, *GGT* gamma-glutamyltransferase, *HDL-c* high density lipoprotein cholesterol, *HGB* hemoglobin, *Hs-CRP* high sensitivity C reactive protein, *LDL-c* low density lipoprotein cholesterol, *Lp-PLA2* lipoprotein-associated phospholipase A2, *Plt* platelet, *RBC* red blood cell, *TBIL* total bilirubin, *Tch* total cholesterol, *TG* triglyceride, *WBC* white blood cell.

### Serum RIs of Lp-PLA2 based on gender and Age

Male serum Lp-PLA2 levels were significantly higher than in females (P < 0.05). Selected participants were divided into different groups according to gender and age (18–29, 30–39, 40–49, 50–59, 60–69, and ≥ 70 years). Serum Lp-PLA2 levels among males in the six age groups did not show statistically significant differences and thus were combined into one group. However, female Lp-PLA2 levels showed statistically significant difference among the six groups (P < 0.05), and post hoc analysis found that there were no statistically significant differences among the three groups < 50 years or the three groups ≥ 50 years of age and thus female participants were combined into two groups (18–49 and 50–88 years). Nonparametric statistical methods were used to calculate RIs with central 95th percentiles, and the 2.5th and 97.5th percentiles of serum Lp-PLA2 levels according to gender and/or age were showed in Table 2. Lp-PLA2 levels in females 18–49 years of age were significantly lower than those of females 50–88 years of age (P < 0.05).

**Table 2:** RIs of serum Lp-PLA2 levels (U/L) in healthy Chinese Han adults

**Age (years)**	***n***	**Median**	**Percentile of reference interval**				
			**2.5**^**th**^	**25**^**th**^	**50**^**th**^	**75**^**th**^	**97.5**^**th**^
**Female**							
18-49	145	415	194	345	415	489	640
50-88	226	462	208	388	462	542	698
**Male**							
18-87	392	504	230	399	504	579	728

### Correlation between Lp-PLA2 and other blood markers

Spearman correlations were utilized to analyze differences between Lp-PLA2 and other blood markers (Table 3), which indicated that serum Lp-PLA2 levels were positively associated with age, BMI, the levels of ALT, GGT, TBIL, TG, Tch, LDL-c, apoB, Glu, and Hs-CRP, as well as HGB, WBC and RBC counts (P < 0.05). Conversely, serum Lp-PLA2 levels were negatively correlated with HDL-c and apoAI levels (P < 0.05), and no correlation was found with Plt counts.

**Table 3:** Spearman correlation coefficients between serum Lp-PLA2 and blood markers

**Marker**	**Correlation coefficient**	**P value**
Age	0.169	<0.001
BMI	0.121	0.025
ALT	0.211	<0.001
GGT	0.199	<0.001
TBIL	0.240	<0.001
TG	0.283	<0.001
Tch	0.336	<0.001
HDLc	−0.186	<0.001
LDLc	0.429	<0.001
apoAI	−0.099	0.006
apoB	0.504	<0.001
Glu	0.093	0.010
Hs-CRP	0.08	0.028
WBC	0.123	0.001
HGB	0.279	<0.001
RBC	0.262	<0.001
Plt	0.012	0.741

*Abbreviations*: *ALT* alanine aminotransferase, *ApoAI* apolipoprotein AI, *ApoB* apolipoprotein B, *BMI* body mass index, *GGT* gamma-glutamyltransferase, *Glu* glucose, *HDL-c* high-density lipoprotein cholesterol, *HGB* hemoglobin, *Hs-CRP* high sensitivity C reactive protein, *LDL-c* low density lipoprotein cholesterol, *Lp-PLA2* lipoprotein-associated phospholipase A2, *Plt* platelet, *RBC* red blood cell, *TBIL* total bilirubin, *Tch* total cholesterol, *TG* triglyceride, *WBC* white blood cell.

## Discussion

This study establishes the RIs of serum Lp-PLA2 levels in healthy Chinese Han participants according to the CLSI C28-A3 guidelines [11]. Furthermore, sample sizes used in this study are in accordance with the guidelines, which recommend a minimum of 120 subjects for each partition group [11]. This study demonstrates gender differences in RIs of serum Lp-PLA2 levels, with, significantly lower serum levels in females as compared to males (230–728 U/L), as well as a female age difference, with females 18–49 years of age showing lower levels those of females 50–88 years of age (194–640 and 208–698 U/L, respectively). These results are in agreement with previous studies reporting lower Lp-PLA2 activity levels in female as compared to male [15,16]. Kosaka *et al.*[17] reported the serum Lp-PLA2 activity for females was significantly lower than that of males at the 5% level in a healthy Japanese population, and suggested that Lp-PLA2 activity levels were inversely proportional to estrogen concentrations, with Lp-PLA2 activity levels in females ≥ 50 years higher than in younger females, similar to the results of this study.

The median levels of Lp-PLA2 in males and females in this study were 504 U/L and 446 U/L, respectively, which were similar to the values (male, 506 U/L; female, 439 U/L) previously reported by Winkler *et al.*[16]. The low Lp-PLA2 levels observed in females may result from the secretion of estrogen, as Lp-PLA2 levels in pre-menopausal females were lower than those of menopausal females. This estrogen effect was suggested in a study by Yoshimura *et al.*[18] showing a 26% reduction in Lp-PLA2 levels in menopausal females two weeks after receiving estrogen replacement therapy.

Lp-PLA2 is an enzyme that mainly exists in combination with lipoprotein particles in blood circulation, with 80-85% bound to LDL, approximately 15-20% bound to HDL [19]. Da Silva *et al.* analyzed the influence of obesity on Lp-PLA2 and found that enzyme activity was positively associated with BMI and the function of Lp-PLA2 changes with adolescent obesity [20]. A positive correlation also had been established between Lp-PLA2 levels and Tch, LDL-c and apoB, and a negative correlation with HDL-c and apoA I, which was confirmed by the results of this study. These data suggest that Lp-PLA2 is present in atherogenic lipoproteins. Similarly, the confirmation of a positive correlation with ALT, GGT and TBIL can be associated with the secretion of Lp-PLA2 from liver cells [21], which may be the principal source of biliary Lp-PLA2 responsible for the adjustment of gastrointestinal PAF and phospholipid metabolism [22]. Stafforini *et al.* found that apoB was the factor most strongly correlated with Lp-PLA2 levels, which manifests a strong bonding force with Lp-PLA2 due to its carboxyl terminus [23]. ApoB is a structural protein of lipoproteins, excluding HDL-c, and plays an important role in transporting lipids to extrahepatic tissues and recognizing LDL receptors. Some studies indicated that Lp-PLA2 induced WBC activation and inflammatory responses *in vitro*, and that this activation may be associated with a rapid increase in circulatory Lp-PLA2 expression [24,25]. Yoshida *et al.* found that Lp-PLA2 may play an important role in scavenging oxidized phospholipids in RBC membranes and in maintaining the normal rheological properties of erythrocytes [26]. Furthermore, Uydu *et al.* reported that serum Lp-PLA2 was positively associated with the variations of Hs-CRP in stable CAD patients and was a more reliable indicator of coronary stenosis [27]. These findings may help explain the positive correlations observed between Lp-PLA2 levels and Hs-CRP, HGB, WBC, and RBC in this study. Taken together, the data suggest that after Lp-PLA2 is bound to LDL via apoB, lysophosphatidylcholine and free oxidized fatty acids, two strong pro-inflammatory factors, are produced through the hydrolysis and oxidation of phospholipids, thereby promoting chronic inflammation and atherosclerosis [28].

## Conclusions

Lp-PLA2 has recently attracted considerable attention for its importance in predicting cardiovascular disease and prognostic evaluation. In addition, Lp-PLA2 has also been proposed as a new independent risk factor for the development and progression of coronary heart disease [29,30]. As a result, the establishment of a reference interval for Lp-PLA2 levels is needed for the clinical evaluation of patients with cardiovascular disease. Moreover, considering the influence of gender and age on Lp-PLA2 levels, our results not only provide an alternative reference for clinical practice, but also pave the way for further clinical studies.

## Competing interests

The authors declare that they have no competing interest.

## Authors' contributions

The study was designed by YC. Experimental data was obtained by LF, GF and YC. Data analyses were performed by GF and LF. The paper was written by LF. All authors read and approved the final version of the manuscript.
